# Safety of adjuvant endocrine therapies in hormone receptor–positive early breast cancer

**DOI:** 10.3747/co.v16i0.457

**Published:** 2009-07

**Authors:** S. Sehdev, G. Martin, L. Sideris, W. Lam, S. Brisson

**Affiliations:** * The Oncology Group, William Osler Health Centre, Brampton, ON; † Hôpital Notre Dame, University of Montreal, Montreal, QC; ‡ Maisonneuve–Rosemont Hospital, University of Montreal, Montreal, QC; § Burnaby Hospital Regional Cancer Centre, Burnaby, BC; || Hôpital de Gatineau, Gatineau, QC

**Keywords:** Adjuvant endocrine therapy, breast cancer, aromatase inhibitors, side effects

## Abstract

Postmenopausal patients with hormone-sensitive early breast cancer are typically treated with adjuvant endocrine therapy, which significantly reduces the risk of recurrence. Because treatment is of a long duration, side effects from adjuvant therapy can be problematic. The aromatase inhibitors (AIS) are replacing tamoxifen as first-line treatment agents for early breast cancer. Here, we present the side-effect data associated with AIS in relation to bone, gynecologic, and cardiovascular health and to arthralgia and myalgia. Although AIS have been shown to decrease bone density, increase arthralgia, and affect vaginal health, these adverse events are usually manageable, and several strategies can be followed to improve quality of life in women on AI treatment. To optimize adherence to therapy. It is important that these issues are addressed so that women can benefit from treatment.

## 1. INTRODUCTION

The selective estrogen receptor (SERM) antagonist tamoxifen and, more recently, the aromatase inhibitors (AIS) anastrozole, exemestane, and letrozole have all been used as adjuvant endocrine therapy agents for the treatment of hormone receptor–positive early breast cancer. Because the presence of circulating estrogen is associated with breast cancer recurrence 1, these therapies are targeted toward reducing or abrogating circulating estrogen.

Long-term treatment with endocrine agents raises issues both for patients and for physicians in balancing the potential benefits of reducing recurrence with the possible side effects. The SERMS and AIS reduce estrogen in different ways. The SERMS bind to the estrogen receptor on tumour cells, thus blocking the ability of the cells to proliferate; however, the SERMS also have estrogenic effects on certain tissues such as bone 2. The AIS, on the other hand, block the ability of the aromatase enzyme to convert androgen precursors in peripheral tissues to estrogen, which is the major source of circulating estrogen in postmenopausal women 3. Both classes of agents result in accelerated symptoms of estrogen depletion, which has an effect on bone, cardiovascular, and gynecologic health and which can also give rise to arthralgia. However, because of their differing modes of action, the SERMS and AIS have different side-effect profiles.

The effects of tamoxifen are well documented and include gynecologic symptoms, an increased risk of endometrial cancer, and an increased risk of deep-vein thrombosis 4. The AIS, because of their profound ability to deplete estrogen, are associated in clinical trials with loss of bone density, musculoskeletal pain, and arthralgia 5. Although the choice of adjuvant agent depends on patient characteristics such as age, comorbidities, and so on, the AIS are increasingly becoming first-line agents based on their efficacy and tolerability profiles. However, it is important for clinicians to be aware of the differential toxicity profiles of these agents as compared with tamoxifen and to be familiar with various strategies for managing the potential side effects. It is also important to consider how toxicity data were obtained, because these data often vary from study to study and may result in misleading interpretations.

For example, to prevent potential bias in event reporting in the Arimidex, Tamoxifen, Alone or in Combination (ATAC) trial, the largest and longest trial evaluating an AI, investigators were required to ask patients at every visit whether they had suffered adverse effects rather to use pre-specified checklists 6. On the other hand, the Breast International Group (BIG) 1–98 trial comparing letrozole with tamoxifen reported adverse events by checking specific boxes on case report forms 7. A decision to specifically record particular events may yield conclusions that are different from those resulting from nonspecific reporting of adverse effects, and advocates of the former approach may argue that “if you do not look, you may not find” 8.

## 2. BONE HEALTH

### 2.1 Background

Typically, bone health may deteriorate in menopausal women, because a decline in estrogen concentration accelerates bone loss 9,10, and women with breast cancer are at added risk. The Women’s Health Initiative Observational Study showed that bone mineral density (BMD) is lower in breast cancer survivors than in matched controls and that osteoporosis is often undiagnosed in these women 11. A recent study also showed that after 29.5 months, lumbar BMD is lower in women who have undergone chemotherapy 12.

Not surprisingly, given the profound estrogen depletion effect of AIS, clinical trials have demonstrated an increased risk of fractures with AI treatment as compared with treatment with tamoxifen (Table I). In those trials, women on anastrozole 6,14,15,18, letrozole 7 or exemestane 17 were all found to have a higher incidence of fracture than were women on tamoxifen, and the incidence varied with the AI and the time to analysis. In contrast, tamoxifen has been associated with increased BMD in postmenopausal women treated for breast cancer, but with decreased BMD in premenopausal women 19.

The ATAC trial included a bone subprotocol involving 81 and 86 women on anastrozole and tamoxifen monotherapy respectively, who either had normal BMD or osteopenia at baseline. At 5 years, no women with normal BMD on either treatment became osteoporotic, but anastrozole was associated with a statistically significant (*p* = 0.0001) decrease in hip and lumbar spine BMD 20. As well, turnover of the bone markers N-terminal telopeptide of type 1 collagen and bone alkaline phosphatase was higher and BMD was lower after 1 year of anastrozole treatment as compared with 1 year of tamoxifen treatment 21,22. Similar results have been reported for letrozole.

The National Cancer Institute of Canada Clinical Trials Group study MA.17B evaluated bone turnover markers and BMD in postmenopausal women randomly assigned to letrozole or placebo after standard adjuvant tamoxifen 23. At 24 months, patients receiving letrozole experienced significant decreases in both total hip and lumbar spine BMD. Similarly, women switched to exemestane following tamoxifen for 2–3 years showed a marked increase in bone turnover markers with a decrease in BMD 24. A substudy of the German Tamoxifen Exemestane Adjuvant Multinational trial also recently showed a decrease in BMD at the spine in women on exemestane; tamoxifen had a protective effect 25.

In healthy postmenopausal women, AIS also decrease estrogen and increase bone markers, as demonstrated in the Letrozole, Exemestane and Anastrozole Pharmacodynamic trial. Changes were comparable with each AI except for exemestane, which increased procollagen type 1 N-terminal propeptide 4 times more than did the other AIS at 24 weeks 26.

### 2.2 Recent Evidence

Current thinking is that the benefits of AI treatment outweigh the risks of bone fracture, because osteoporotic therapy can be simultaneously given and has recently been shown to be highly effective. In the Study of Anastrozole with the Bisphosphonate Risedronate (SABRE), 234 women on anastrozole concurrently treated with the bisphosphonate risedronate showed an increase in BMD and a decrease in bone turnover markers at 12 months as compared with results in women on anastrozole alone 27. Similarly, in another double-blind randomized placebo-controlled study to evaluate the effect of a bisphosphonate in women on anastrozole (the ARIBON trial), monthly ibandronate was associated with bone loss prevention and increases in BMD at the lumbar spine and hip in osteopenic and osteoporotic patients at 2 years 28.

In the Zometa–Femara Adjuvant Synergy Trial, concomitant administration of zoledronic acid in 522 women started on letrozole (“upfront group”) resulted in significantly higher lumbar spine and hip BMD at 24 months than was seen in the 538 women who were given the bisphosphonate only if their T-score fell to −2 or lower during therapy, or if spinal fracture was evident at 36 months (8.2% vs. 4.7% respectively) 29. Predictably, fewer fractures occurred in women given zoledronic acid up front (2.5% vs. 3.2%). Similarly, a substudy of the International Breast Cancer Intervention Study showed that, although women on anastrozole experienced significant bone loss as compared with losses seen in women on placebo, women who joined the study with an osteopenic or osteoporotic T score gained BMD after receiving risedronate treatment for 1 year 30.

In premenopausal women, treatment with goserelin plus tamoxifen or anastrozole for 3 years was associated with significant bone loss, and bone was still compromised after 5 years, particularly in women who had been on anastrozole. However, 4 mg zoledronic acid given every 6 months for 3 years completely inhibited bone loss and led to improved BMD at 5 years 31. Similarly, in the 100-month evaluation of the ATAC trial, though fracture rates overall were higher in women on anastrozole during the treatment period, the reported fracture incidence was the same after treatment had been completed 15; a recovery of BMD was noted at the lumbar spine and a slowing in loss at the hip was observed in women on anastrozole after completion of treatment 32.

### 2.3 Management

Bone loss should be a consideration in women on AI treatment, and regular screening is recommended. The American Society of Clinical Oncology (ASOC) recommended in 2003 that all women at high risk of osteoporosis, including women on AIS, undergo baseline assessment of BMD and annual monitoring while on treatment for breast cancer 33. However, a suggestion has since been made that regular monitoring be carried out only on patients at risk for osteoporosis 34 because, in the ATAC trial, no women with normal BMD at baseline had developed osteoporosis at 5 years 13, and BMD appeared to recover or slow once patients stopped treatment 32. Risk factors include an age of 60 years or older, cigarette smoking, steroid use, family history of osteoporosis, and a low body mass index 35. To prevent bone loss, lifestyle changes that can improve or maintain bone health, such as increased exercise, should be discussed with the patient before the start of AI therapy 36. Data from recent trials (as described earlier) indicate that, when necessary, bisphosphonate therapy can prevent further bone loss, so that patients can continue with AI therapy.

## 3. ARTHRALGIA

### 3.1 Background

Joint and musculoskeletal pain increase with age in women, reaching a peak during menopause and postmenopause, suggesting that symptoms may be related to estrogen depletion 37,38. In clinical trials, the AIS are associated with a higher incidence of joint symptoms than tamoxifen treatment is (Table II), with a variation in incidence with the various AIS, likely because of different data-gathering methods.

If pain occurs, it seems to manifest shortly after the initiation of treatment. For example, in the ATAC trial, arthralgia was most common in the first 6 months after the start of treatment 13. However, for most patients in the ATAC trial, symptoms resolved within 18 months of starting treatment, and of the patients who recovered, half were symptom-free within 6 months of onset 41.

### 3.2 Recent Evidence

A substudy of the ATAC trial revealed that risk factors for developing joint symptoms were prior use of hormone replacement therapy, hormone receptor positivity, obesity, prior chemotherapy, and treatment with anastrozole, leading to significant symptom increases of 12.3%, 8.2%, 6.8%, 5.9%, and 5.7% respectively 42. In a small study of 170 women with invasive estrogen receptor–positive breast cancer, who were treated with either anastrozole for 12 weeks followed by letrozole (or the reverse) and who were subsequently switched to tamoxifen, joint pain was reported by 76% while on an AI, and no difference between the two AIS was observed 43. Interestingly, in that study, 50% of the patients with joint symptoms on one AI did not have symptoms when switched to the other AI, and 75% of the patients who had symptoms on an AI did not have symptoms on tamoxifen. These data indicate the possibility of switching AIS should joint pain be problematic.

An association between persistent musculoskeletal pain and vitamin D hypovitaminosis has been reported 44. In a recent study, women with invasive breast cancer who were started on letrozole together with vitamin D supplementation (50,000 IU weekly) showed improvement in joint symptoms and fatigue 45. Interestingly, in that study, 63% of women initiating AI therapy had vitamin D insufficiency (less than 32 ng/mL).

### 3.3 Management

**FIGURE 1:**
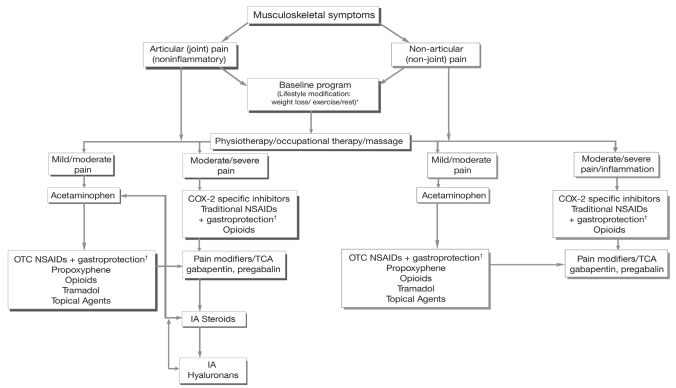
Management of musculoskeletal symptoms in patients with early breast cancer taking an aromatase inhibitor 47. OTC = over-the-counter; COX-2 = cyclooxygenase 2; NSAID = nonsteroidal anti-inflammatory drug; TCA = tricyclic antidepressant; IA = intra-articular. ^†^ Misoprostol or proton pump inhibitor.

The prevalence of arthralgia in patients on AIS can be significant. In a small study of 56 patients who were not in clinical trials but who were receiving AIS, arthralgia or bone pain or both were reported in 61% and resulted in discontinuation of the drug in 20% 46. Thus, adequate and prompt management of pain is required. Arthralgia associated with AI treatment is usually manageable and has been comprehensively dealt with by Thorne, with an algorithm for diagnosis and treatment (Figure 1) 47. Briefly, the following steps are advocated:

- Physical examination and patient history are required to rule out pain from osteoarthritis, rheumatoid arthritis, sleep disturbance, and so on.
- Articular pain should be distinguished from nonarticular pain, and inflammatory from non-inflammatory pain.
- The most common sites of arthralgia are the knees, hands, wrists, and shoulders.
- The most appropriate intervention for pain management may be a combination of lifestyle changes—for example, exercise, and calcium and vitamin D supplements—in conjunction with pharmacologic interventions.
- Drug therapy includes the use of acetaminophen, nonsteroidal anti-inflammatory drugs, opiates, glucosamine, and topical medications such as capsaicin or methylsalicylate.
- Patients with severe pain should be referred to a rheumatologist.
- Pain from bone metastases should be ruled out by history and physical examination or, where appropriate, imaging studies.
- A drug holiday of 3–4 weeks can also be helpful in confirming the cause of the pain. Anecdotally, rechallenge with the same AI is often well tolerated, but if symptoms significantly affect quality of life or impair activities of daily living, a switch to another endocrine agent can be considered.

## 4. GYNECOLOGIC HEALTH

### 4.1 Background

As compared with placebo, tamoxifen treatment is associated with an increased incidence of vaginal bleeding, endometrial polyps, endometrial thickening, and ovarian cysts. Prolonged use is associated with significant gynecologic complications, including a doubled to tripled risk of endometrial cancer 4. By comparison, quality-of-life assessments in the ATAC trial reported that, as compared with women on tamoxifen, women on anastrozole experienced fewer hot flashes, vaginal bleeding, and vaginal discharge 13 (Table III), but more vaginal dryness, painful intercourse, and loss of sexual interest 48. The risk of endometrial cancer was in women on anastrozole was one quarter that seen in women on tamoxifen (0.2% vs. 0.8% respectively) 13.

As compared with women on tamoxifen, women on letrozole in the BIG 1-98 study also suffered significantly less vaginal bleeding and fewer hot flashes (Table III). However, in the Intergroup Exemestane Study (IES), in which women were switched to exemestane after 2–3 years on tamoxifen, more women on exemestane experienced hot flashes; however, the difference was not statistically significant 17.

As compared with the 60-month analysis 13, the 100-month analysis 15 of the ATAC trial showed no difference in gynecologic symptoms, except for a higher incidence of endometrial cancer with tamoxifen than with anastrozole (Table III)—an incidence that persisted when treatment was completed 15. In the IES trial, analysis of data 5 years post randomization showed that hot flushes and night sweats remained problematic not only for women who had been switched to exemestane, but also for those who remained on tamoxifen; however, severe vaginal discharge was significantly higher for those on tamoxifen 49.

Estrogen levels in women on AI treatment are so low that their response to estrogen may be heightened, and the effect of this heightened response on breast cancer recurrence is unknown. In a prospective study, 7 postmenopausal women on AI treatment who were started on a vaginal estrogen tablet for severe symptoms of atrophic vaginitis showed significantly raised serum estradiol levels within 2 weeks 50. Because the efficacy of AIS is based on completely depleting estrogen to prevent proliferation of tumour cells, that effect might be counteracted with increases in systemic estrogen 51. However, in another small study, 5 of 12 women on AI treatment concurrently treated with vaginal estrogen for 3 months experienced no increase in serum estrogen levels (the remainder did), suggesting that a subset of women on AI therapy may be able to use vaginal estrogen to treat vaginal atrophy 52. In the absence of a definitive link between topical estrogen therapies and breast cancer prognosis, the Society of Obstetricians and Gynaecologists of Canada suggest that women with a history of breast cancer first consider non-hormonal options to treat symptoms of vaginal atrophy, but that local intravaginal estrogen may be considered for women with low risk of recurrence if non-hormonal options are ineffective and quality of life becomes an issue 53.

### 4.2 Management

Lifestyle modifications such as the use of layered clothing—or even acupuncture, meditation, and biofeedback—have been suggested for alleviating hot flashes in women on adjuvant hormone therapy 54. If symptoms are severe, selective serotonin reuptake inhibitors such as venlafaxine, sertraline, and paroxetine are recommended 54. As compared with placebo, venlafaxine, even at 37.5 mg, can considerably reduce hot flashes 55. However, tamoxifen is converted to its active metabolite endoxifen by the cytochrome 450 enzyme CYP2D6 56 and venlafaxine has been found to be a weak inhibitor of CYP2D6 (paroxetine is a potent inhibitor) 57. Thus, in women who are genetically predisposed to show less-effective CYP2D6 metabolism, possible drug interactions should be assessed 58.

Management of sexual dysfunction in postmenopausal women on AI therapy has recently been reviewed by Derzko *et al.* 59. Briefly, the following steps are advocated in women with sexual dysfunction:

- A sexual history, physical examination, and hormonal evaluation should be carried out.
- Therapeutic interventions should be tailored to address each area of distress.
- Non-hormonal treatments are the first-line recommendation for urogenital atrophy, vaginitis, and dyspareunia.
- Vaginal dryness may be treated with lubricants.
- Low-dose local vaginal estrogen therapy may be considered for highly symptomatic patients who are unresponsive to non-hormonal therapy, but this approach is controversial.

## 5. CARDIOVASCULAR HEALTH

### 5.1 Background

Mortality in breast cancer survivors occurs not only because of cancer recurrence, but also because of cardiovascular (CV) disease (death from other causes has not been found to be significantly elevated) 60. Thus, it is important to evaluate the CV effects of agents used for adjuvant endocrine treatment, particularly in women at risk.

In the ATAC trial, no difference was observed in the occurrence of ischemic CV events between women on tamoxifen and those on anastrozole, and the most common event was angina 6. Alterations in lipid profile, such as increases in cholesterol, triglycerides, and low-density lipoprotein (LDL) cholesterol, and decreases in high-density lipoprotein (HDL) cholesterol are risk factors for the development of CV disease. Tamoxifen has been shown to decrease LDL by 20% and total cholesterol by 12% in disease-free postmenopausal women after 2 years, with the effect being maintained after 5 years of treatment 61,62. Similar effects were found in postmenopausal node-negative breast cancer patients on tamoxifen 63. The AIS, on the other hand, have shown varying effects on serum lipid levels. Some studies show anastrozole having little effect on serum lipids; others show hypercholesterolemia 64. Although lipid concentrations were not routinely assessed in the ATAC trial, women on anastrozole showed a higher incidence of hypercholesterolemia than did those on tamoxifen 6.

A similar situation has held for letrozole: conflicting data on lipid levels have been seen. In the BIG 1-98 trial, analysis at 51 months showed hypercholesterolemia (predominantly grades 1 and 2) in 51% of women on letrozole as compared with 25% of women on tamoxifen (*p* < 0.001, Table IV) 39. However those data might be misleading, because cholesterol levels (non-fasting) were analyzed every 6 months, and even a single high reading was reported as an event 7. Furthermore, the median changes were not substantially different from baseline levels, whereas baseline levels declined in women on tamoxifen 7, suggesting that the difference between the letrozole and the tamoxifen groups may have been more reflective of the cholesterol-lowering effect of tamoxifen 63. Although the overall incidence of cardiac events did not vary between the two groups at 51 months, a trend toward higher-grade cardiac events was observed in women on letrozole 65. A substudy of the MA.17 trial specifically looking at lipid levels showed that, as compared with placebo, letrozole did not significantly alter serum cholesterol, HDL cholesterol, LDL cholesterol, or triglycerides in non-hyperlipidemic postmenopausal women with primary breast cancer treated for up to 36 months after having received at least 5 years of adjuvant tamoxifen therapy 66.

Trials of exemestane for advanced breast cancer have also yielded conflicting results. One study showed a reduction in cholesterol and total triglycerides, but also an unfavourable reduction in HDL; and another trial showed no lipid changes 67.

It is important to remember that lipid levels are surrogate endpoints and do not necessarily have an effect on CV disease. Despite the favourable effect of tamoxifen on lipid profiles, a meta-analysis of tamoxifen trials showed no effect on the incidence of myocardial infarction (MI), though death from MI was decreased in women likely to have hyperlipidemia and coronary artery disease 68. Also, tamoxifen is associated with an increased incidence of stroke and an increased risk of venous thromboembolism 68. In the ATAC trial, anastrozole was associated with a lower incidence of thromboembolic and cerebrovascular events than was tamoxifen 13 (Table IV). Interestingly, the Italian Tamoxifen Anastrozole trial showed little difference between tamoxifen and anastrozole in terms of CV diseases (6.2% and 7.6% respectively) at 64 months 69. A statistically significant (*p* < 0.001) lower incidence of thromboembolic events was observed with letrozole as compared with tamoxifen in the BIG 1-98 trial 7,39. However, compared with placebo, letrozole showed no statistically significant difference in terms of thromboembolic events, MI, stroke, or angina. In the IES trial, in which women were switched from tamoxifen to exemestane after 2–3 years, exemestane was associated with an increase in MI (1% vs. 0.4% with tamoxifen, *p* = 0.02); however, this increase was considered to be statistically nonsignificant, because the cut-off for significance was *p* = 0.01 17.

### 5.2 Recent Evidence

The SABRE study, which was designed to assess the efficacy of concurrent administration of risedronate with anastrozole on bone health, also monitored lipid levels. After 12 months, LDL was decreased, HDL was increased, and no change was observed in total cholesterol both in women on anastrozole alone and in those concurrently treated with the bisphosphonate 27. The 100-month evaluation of the ATAC trial showed no difference in the incidence of MI between women on anastrozole and those on tamoxifen, and a decreased incidence of cerebrovascular events with anastrozole (Table IV) 15. Similarly, in the 5-year analysis of the BIG 1-98 trial, although hypercholesterolemia was evident in twice as many (51%) women on letrozole as on tamoxifen (26%), no difference was observed in cardiac events between the two groups, and a decreased incidence (*p* < 0.001) of thromboembolic events was observed in women on letrozole 70. Furthermore, safety data from several major clinical trials were recently reviewed to assess CV adverse events among the AIS, and CV profiles were found to be similar among women on AIS (as compared with profiles in women on tamoxifen) 71. However, it was recognized that, because of the lack of long-term data, clinical cardiac outcomes and lipid profiles should be closely monitored in patients taking an AI.

### 5.3 Management

No current evidence suggests that the AIS have a particular adverse effect on CV health. As noted in the clinical trials, differences in lipid levels, as compared with levels in women taking tamoxifen, may have more to do with the protective effect of tamoxifen on lipid levels than with an adverse effect of the AIS 51. Patients receiving endocrine treatment should undergo a CV risk assessment and their blood pressure, cholesterol levels, and similar parameters should be monitored as part of a routine health check; however, no specific management strategies are required 51. As is the case for healthy individuals, women are encouraged to exercise and quit smoking.

## 6. CONCLUSIONS

Many of the side effects associated with the AIS stem from their accelerated effect on estrogen depletion, but in general, these agents are well tolerated. So far, no current evidence shows that AIS might be associated with a serious adverse event such as the increased incidence of endometrial cancer noted with tamoxifen treatment. However, although the AIS have been shown to have a negative effect on bone density and arthralgia, these effects are manageable, and interventions can be introduced to alleviate symptoms and prevent unwanted sequelae. Possible side effects should be discussed with the patient, and patient well-being should be monitored for the duration of treatment. Further, patients with known risk factors such as osteopenia, joint pain, or cardiovascular symptoms should be identified at the start of treatment, and appropriate interventions should be applied such that AI treatment can be continued without worsening of symptoms. The goal is to maintain patients on treatment for the allotted length of time while ensuring a comfortable quality of life.

## Figures and Tables

**TABLE I:** Fracture rates with tamoxifen and aromatase inhibitor treatment

*Study^a^*	*Fracture rates (%) with*				p* Value*	*Comment*
	*Tamoxifen*	*Anastrozole*	*Letrozole*	*Exemestane*		
ATAC						
At 60 months^13^	7	11			<0.0001	Age and geographic location added risk for fracture^16^.
At 100 months 14,15	1.9	2.9			<0.0001	
Treatment completed	1.51	1.56			0.79	
BIG 1–98						
At 25.8 months^7^	4		5.7		<0.001	
At 51 months^8^	5.8		8.6		<0.001	
IES^17^	2.3		3.1		0.08	Patients switched to exemestane after 2–3 years on tamoxifen.

a Because of differing methodologies, direct comparisons should not be made between the studies.

ATAC = Arimidex, Tamoxifen, Alone or in Combination; BIG = Breast International Group; IES = Intergroup Exemestane Study.

**TABLE II:** Incidence of arthralgia with tamoxifen and aromatase inhibitor treatment

	*Study*	*Incidence of arthralgia (%) with*				p* Value*	*Comment*
		*Tamoxifen*	*Anastrozole*	*Letrozole*	*Exemestane*		
ATAC	At 60 months^13^	29.4	39.6			0.0001	
BIG 1–98	At 51 months^39^	13.5		20		<0.001	
MA.17^40^		21		25		0.001	Women switched to letrozole following 5 years of tamoxifen. More women experienced myalgia than experienced arthritis.
IES^17^		3.6			5.4	0.01	Women switched to exemestane following 2–3 years of tamoxifen.

ATAC = Arimidex, Tamoxifen, Alone or in Combination; BIG = Breast International Group; IES = Intergroup Exemestane Study.

**TABLE III:** Incidence of gynecologic symptoms in women on aromatase inhibitors (AI) or tamoxifen (TAM)

	*Trial*	*Treatment arm*	*Incidence (%) of*							
			*Hot flashes*		*Vaginal bleeding*		*Vaginal discharge*		*Endometrial cancer*	
			*AI*	*TAM*	*AI*	*TAM*	*AI*	*TAM*	*AI*	*TAM*
ATAC	At 60 months^13^	Anastrozole vs. tamoxifen	35.7	40.9	5.4	10.2	3.2	13.2	0.2	0.8
			*p*<0.0001		*p*<0.0001		*p*<0.0001		*p*=0.02	
	At 100 months^15^	Anastrozole vs. tamoxifen	NR	NR	NR	NR	NR	NR	0.03	0.13
BIG 1-98	At 25.8 months^7^	Letrozole vs. tamoxifen	33.5	38	3.3	6.6	NR	NR	0.1	0.3
			*p*<0.001		*p*<0.001				*p*=0.18	
	At 51 months^39^	Letrozole vs. tamoxifen	32.8	37.4	3.8	8.3	NR	NR	0.16	0.65
			*p*<0.001		*p*<0.001				NR	
IES^17^		Exemestane following 2–3 years tamoxifen vs. tamoxifen	42	39.6	4	5.5	NR			NS
			*p*=0.082		*p*=0.087					

ATAC = Arimidex, Tamoxifen, Alone or in Combination; NR = not reported; BIG = Breast International Group; IES = Intergroup Exemestane Study; NS = nonsignificant.

**TABLE IV:** Incidence of cardiovascular and cerebrovascular events in women on aromatase inhibitors (AIS) and tamoxifen (TAM)

	*Trial*	*Treatment arms*	*Incidence (%) of*							
			*Hypercholesterolemia*		*Cardiovascular disease*		*Cerebrovascular events*		*Thromboembolic events*	
			*AI*	*TAM*	*AI*	*TAM*	*AI*	*TAM*	*AI*	*TAM*
ATAC	At 60 months^13^	Anastrozole vs. tamoxifen	NR	NR	4.1	3.4	2.0	2.8	4.5	6.9
					*p*<0.1		*p*<0.03		*p*<0.03	
	At 100 months^15^	Anastrozole vs. tamoxifen	NR	NR	0.27^a^	0.27^a^	0.16	0.28	NR	NR
BIG 1-98	At 25.8 months^7^	Letrozole vs. tamoxifen	43.6	19.2	6.8	5.6	1.0	1.0	1.5	3.5
							*p*=0.91		*p*<0.001	
	At 51 months^39^	Letrozole vs. tamoxifen	50.6	24.6	9.5	7.5	1.4	1.4	2.0	3.8
			*p*<0.001						*p*<0.001	
IES^17^		Exemestane following 2–3 years tamoxifen vs. tamoxifen	42	39.6	42.6^b^	39.2^b^	NR	NR	1.0	1.9
			*p*=0.082		*p*=0.016				*p*=0.005	

a Incidence of myocardial infarction.

b Not including myocardial infarction.

ATAC = Arimidex, Tamoxifen, Alone or in Combination; NR = not reported; BIG = Breast International Group; IES = Intergroup Exemestane Study.
